# HTLV-1 and ATLL: Epidemiology, Oncogenesis, and Opportunities for Community-Informed Research in the United States

**DOI:** 10.3390/v17101333

**Published:** 2025-09-30

**Authors:** Adrian Altieri, Sean Patrick Reilly, Abu Mansalay, Alan Soo-Beng Khoo, Nettie Johnson, Zafar K. Khan, Amy Leader, Pooja Jain, Pierluigi Porcu

**Affiliations:** 1 Sidney Kimmel Medical College, Thomas Jefferson University, Philadelphia, PA 19107, USA; 2 Sidney Kimmel Comprehensive Cancer Center (SKCCC), Thomas Jefferson University Hospital, Philadelphia, PA 19107, USA; 3 Department of Medical Oncology, Thomas Jefferson University, Philadelphia, PA 19107, USA; 4 African Cultural Alliance of North America (ACANA), Philadelphia, PA 19143, USA; 5 Department of Microbiology and Immunology, Drexel University College of Medicine, Philadelphia, PA 19104, USA

**Keywords:** HTLV-1, retroviruses, oncogenic viruses, infection and cancer

## Abstract

Human T-cell leukemia virus type 1 (HTLV-1), the first oncogenic human retrovirus, causes adult T-cell leukemia/lymphoma (ATLL), an aggressive neoplasm of mature CD4+ T-cells that is incurable in most patients and is associated with a median survival of less than 1 year. HTLV-1 also causes inflammatory disorders, including HTLV-associated myelopathy/tropical spastic paraparesis (HAM/TSP) and uveitis. The estimated lifetime risks of ATLL and HAM/TSP in HTLV-1 carriers are 3–5% and 0.25–1.8%, respectively. Although there is uncertainty about other health effects of HTLV-1, a recent meta-analysis showed an association between HTLV-1 and cardiovascular, cerebrovascular, and metabolic diseases and a 57% increased risk of early mortality in HTLV-1 carriers, independent of ATLL or HAM/TSP. Furthermore, emerging studies in endemic areas show that outcomes for common cancers, such as cervical cancer and lymphoma (non-ATLL), are inferior in HTLV-1 carriers compared to publicly reported data. Thus, the impact of HTLV-1 may be greater and more diverse than currently understood. This review provides an outline of the prevalence and impact of HTLV-1 and associated disorders in the US, focused on—but not limited to—ATLL, with an emphasis on the social determinants of health that can affect the success of screening and prevention strategies. We also discuss the mechanisms by which HTLV-1 drives the pathogenesis of ATLL and potential strategies for early diagnosis and intervention. Finally, we conclude by suggesting approaches to designing and implementing community-informed research initiatives in HTLV-1 and ATLL.

## 1. Introduction

Human T-cell lymphotropic virus 1 (HTLV-1), a deltaretrovirus member of the *Retroviridae* family, was identified by Robert Gallo and colleagues in 1980 as the only known oncogenic human retrovirus [1]. Several HTLV subtypes have been discovered (HTLV-2, HTLV-3, and HTLV-4), but only HTLV-1 has been definitively linked to human cancer. HTLV-1 causes adult T-cell leukemia/lymphoma (ATLL), a highly aggressive and often incurable mature T-cell malignancy [2,3]. HTLV-1 is also associated with non-neoplastic but highly debilitating chronic inflammatory diseases, including HTLV-1-associated myelopathy/tropical spastic paresis (HAM/TSP) and uveitis [4,5].

HTLV-1 has a heterogeneous distribution worldwide, with higher seroprevalence rates in southwestern Japan, parts of South America, the Caribbean, the Middle East, Australo-Melanesia, and Western and sub-Saharan Africa [6,7,8]. It is estimated that 10 to 20 million individuals worldwide are infected with HTLV-1 [9]. While the overall seroprevalence of HTLV-1 in the United States, based on blood donor screening, is low (<1%), the estimated prevalence in the general US population, and, in particular, in cities with large immigrant communities from endemic areas, is unknown but likely higher [6,9,10].

Several horizontal and vertical modes of HTLV-1 transmission have been described, although the relative frequency of each mode of transmission is not clearly established, and the factors affecting the risk of transmission are not fully known [8]. Epidemiological data suggests that the most common routes of transmission may differ in various populations and geographical areas. Sexual transmission has been reported as common in Brazil, Central Australia, and in non-endemic areas. The transfusion of HTLV-1-infected blood is a frequent mode of infection in countries where blood donors are not screened, such as parts of Africa and South America. Needle sharing among drug users is a recognized mode of horizontal transmission [11,12]. While horizontal transmission is the primary method of viral spread in developing countries, vertical transmission through breastfeeding from an infected mother used to predominate in Japan, but there is a concerning trend for increasing horizontal transmission within Japan [13,14,15]. There is also a high risk of infection following organ transplantation from an infected donor [16]. While sexual transmission and infection through shared contaminated needles are well described in many American cities, often in the context of coinfection with other viruses (HIV, HBV), the primary mode of the transmission of HTLV-1 in the US is not known.

An important trait of HTLV-1 infection is the virus’s ability to persist as a latent provirus for decades without causing clinical symptoms. Therefore, the majority of those infected remain asymptomatic and unaware that they have contracted the virus, thus contributing to viral transmission and new infections. Over the course of their lifespan, however, approximately 5% and 0.25–3.8% of HTLV-1-seropositive individuals develop ATLL and HAM/TSP, respectively, with a magnitude of risk that depends in part on the geographic location and other factors.

## 2. Epidemiology of HTLV-1 Infection

As a virus with a multi-decade-long latency period that is often asymptomatic, HTLV-1 presents many challenges for epidemiologists. Currently, it is estimated that there are between 10 and 20 million individuals infected with HTLV-1, mostly in the endemic regions of Japan, Africa, Brazil, the Caribbean, and Australia [9]. However, pervasive underreporting and fragmentary epidemiological data have resulted in most studies being based in the first of these regions, largely neglecting the other ones.

### 2.1. Japan

While both Australia and Japan are considered “developed” countries, HTLV-1 in the former is predominantly concentrated in the rural, indigenous, and economically disadvantaged areas of central Australia, drawing a sharp contrast to the significant presence of the virus in the wealthy, highly populated regions of Kyushu-Okinawa, Kinki, and Kanto in Japan [17,18]. However, the uneven distribution of viral burden that is observed in all endemic areas seems to persist in Japan. Mostly concentrated in these “pockets” of high HTLV-1 seroprevalence, an estimated 1.08 million people were carriers of HTLV-1 in 2006–2007. An updated figure from 2016 held that the incidence of the virus was approximately 3.8 per 100,000 person-years, along with around 4000 new infections yearlong among adults and adolescents [17]. The age at which an individual contracts HTLV-1 is noteworthy; the average age at which ATLL develops is significantly higher in Japan than in other endemic countries at 67.5 years old in 2010–2011 [17]. As a nation with a negative birth rate and an increasingly aging population, contracting the virus during adolescence (as opposed to shortly after birth through breastfeeding) may result in a later age of ATLL onset.

Despite the clear trend of HTLV-1 infection targeting vulnerable populations, of the 2100 new ATLL cases diagnosed globally in 2012, 71.4% of patients lived in developed countries, while the remaining 28.6% lived in developing countries [17]. HTLV-1 infection is necessary but not sufficient for oncogenesis in ATLL, and several associated risk factors have been identified. For instance, the epidemiological data from Japan demonstrates that chronic immunosuppression is a driving risk factor in ATLL development in developed countries. Specifically, intensive and prolonged immunosuppression in patients with renal or liver transplantation, Sjögren’s syndrome, or rheumatoid arthritis has been recognized as an important pathogenetic factor in the transition from an asymptomatic HTLV-1 carrier status to ATLL, as suggested by Yoshizumi et al. in an in-depth analysis of Japanese cohorts [19,20]. Chronic parasitic infections, particularly those caused by *S. stercoralis*, have also been implicated in progression to ATLL by inducing the polyclonal expansion of HTLV-1-infected CD4^+^ T-lymphocytes [21]. As the lone country with HTLV-1 endemic to developed areas, Japan will continue to serve as a model to improve on the currently incomplete knowledge of the risk factors of viral transmission and ATLL oncogenesis in developed countries.

### 2.2. Brazil

Currently, epidemiological studies focused on HTLV-1 prevalence in Brazil are primarily focused on the burden of the virus on vulnerable groups and on the development of non-ATLL HTLV-1-associated diseases. With a current estimate of 800,000–2.5 million current HTLV-1 carriers in Brazil, many seropositive individuals suffer from HAM/TSP, which can leave up to 50% of those affected wheelchair-bound, along with infective dermatitis, arthritis, keratoconjunctivitis, and several pulmonary pathologies [7]. While ATLL has been well documented in Brazil, prevalence estimates remain difficult to come by—a recent retrospective study found that ATLL accounted for ~16% of mature T-cell neoplasms (MTCNs) in Brazil, which can be contrasted to ~1% in Europe and 2% in North America with three-year overall survival rates in the cohort of South American countries of 25% [22]. Aggressive disease (lymphomatous and acute subtype) was diagnosed in 85% of patients. To date, most research efforts have been focused on the development of public health policies designed to slow the spread of the virus by targeting the most common vectors of viral transmission. The implementation of these public mitigation strategies in a resource-constrained environment such as Brazil suggests that a combination of community awareness, proper education, and relatively straightforward compliance requirements may provide an actionable model for other low-socioeconomic and vulnerable communities especially at risk of greater HTLV-1 incidence [23].

HTLV-1 can be sexually transmitted, and the transmission of the virus, primarily from semen, has been targeted as an important factor to control. Beyond barrier methods during sexual activity, required screening tests for sperm donors and recipients of assisted reproduction, such as in vitro fertilization and the addition of HTLV-1 seropositivity to exclusion criteria in sperm donation, are current measures in place that seem to have curtailed the spread of HTLV-1 in Brazil. Furthermore, complementary efforts for the screening of sexual contacts, regular counseling on HTLV-1, the inclusion of HTLV-1 on sexually transmitted illness (STI) panels, and general awareness campaigns are ideally suited to further limit the spread of the virus through sexual contact or genital fluids [23].

Current and proposed policies in Brazil have also been designed to mitigate the spread of HTLV-1 transmission through parenteral means, as well as breastfeeding. As in Japan, solid organ transplantation is a major vector of HTLV-1 transmission, with a reported staggering figure of a 100% risk of infection upon receiving a solid organ transplant from an HTLV-1-positive donor along with a 40% risk of developing HAM [24,25,26]. Since 1993, donated blood products in Brazil have been screened for HTLV-1, with infection being considered an exclusion criterion. It has been widely reported that HTLV-1 infection is increasingly prevalent among people who inject drugs (PWIDs) [27]. In this context, it has been proposed that policies governing needle exchange programs, sterile needle distribution, and safe disposal for used needles in areas with high injection drug use rates may help curtail the spread of HTLV-1 [23].

Beyond parenteral transmission, vertical mother-to-child transmission through breastfeeding makes up 14.1% of the total HTLV-1 cases in urban areas of Brazil. The situation is even more dire in underserved rural indigenous populations, with a 30% rate of transmission in the Amazon region of Brazil [28,29]. Following the identification of infected women, the current recommendations of the Brazilian Ministry of Health include the cessation of breastfeeding, pharmaceutical therapy to stop lactation, the distribution of milk alternatives (such as formula), and antenatal screening during the first trimester of pregnancy. Despite these initiatives, low-income women with a high proviral load (PVL) in their blood and milk continue to be at the highest risk of spread. Longer periods of breastfeeding, HLA concordance between mother and fetus, and coinfection with *S. stercoralis* serve as compounding risk factors for mother-to-child spread [30].

Furthermore, the experience of endemic viral transmission in Brazil validates the premise that HTLV-1 infection is a neglected disease that disproportionately affects neglected populations. Several demographic subsets within Brazil were found to display the greatest vulnerability to HTLV-1-associated morbidities and mortality, namely those of black or mixed ethnicities, of low socioeconomic status, of low education level, and of the female gender, especially those who are victims of domestic and/or sexual violence, concepts that must remain at the forefront when considering informed mitigation strategies in other at-risk groups [23,31].

### 2.3. Africa

Western, Central, and Southern Africa are endemic regions for HTLV-1. With a markedly heterogeneous distribution, approximately 0.3–3% of the adult population is seropositive in these highly endemic areas [7,32]. As has been noted in other areas, such as Japan, there are “clusters” of high HTLV-1 seroprevalence with surrounding areas of little-to-no HTLV-1 burden. The prevalence of HTLV-1 increases with age and is the highest among women, with up to 6.8% and 7.7% seropositivity in pregnant women living in West and Central Africa, respectively [7,32,33]. Beyond infection in pregnant women, HTLV-1 is particularly prevalent in blood donors in central Africa, making up to 6% of the donor population in East Gabon, for instance [7]. The dominant subtype of HTLV-1 also greatly depends on region—HTLV-1A is seen the most in West and North Africa, while HTLV-1B predominates in the center of the continent. The dominance of each subtype is quite pronounced in each region—other minor subtypes are exceedingly rare [7].

A marked lack of access to resources has also been found to impact nearly every mode of viral transmission. For example, the absence of standardized and systematic screening for HTLV-1 in African blood banks has led to the spread of the virus in infected blood products to the point that the risk of contracting the virus from transfusions rivals that of mother-to-child transmission [34]. In addition, a lack of access to breast milk alternatives has allowed for an almost unchecked vertical transmission of HTLV-1 in Central Africa, with rates of transmission being as high as 22% in Gambia and 25% in Guinea-Bissau [35,36].

Due to massive underreporting, the true incidence of ATLL and HAM/STP in endemic areas of Africa remains unknown. There is a notable shortage of locally reported instances of these diseases—most cases in African people are documented in medical returnees or immigrants in the United Kingdom, France, and the United States. In addition, the reporting of ATLL is particularly deficient due to its rarity and lack of awareness on the part of local clinicians, including hematologists.

As the largest area in which HTLV-1 is endemic, the African continent faces many difficulties in both controlling the spread of the virus and documenting infection. Data concerned with the spread of HTLV-1 in Africa remains fragmentary with very few large-scale, representative epidemiological studies performed. As in Brazil, many HTLV-1 studies are performed based on serology without confirmatory tests. Sub-Saharan Africa faces a unique problem due to the common finding of indeterminate seroreactivity patterns in local populations, leading to difficult-to-interpret results or even false positives [37]. The majority of studies were also performed on the at-risk populations of blood donors and pregnant women [38]. The results of such studies are difficult to generalize to the broader population. African populations are no exception in presenting the challenge of estimating the overall prevalence of HTLV-1, as progressive changes in commonly used assays or the use of entirely new assays altogether preclude the estimation of an interpretable and accurate figure of the population currently carrying the virus. In sum, African epidemiological data are vastly incomplete, and a widespread lack of access to resources of all kinds, from baby formula to proper diagnostic and confirmatory tests, represents an immense obstacle to controlling and documenting the spread of the virus. 

### 2.4. United States

With few exceptions, very limited information is currently available about the distribution and seroprevalence of HTLV-1 infection across the United States. Earlier studies suggested that areas with larger proportions of immigrants from endemic areas, particularly the Caribbean, may be especially at-risk for HTLV-1 infection. For instance, a study reported that the prevalence of HTLV-1 in central Brooklyn was comparable to that of the Caribbean islands. A yearlong pilot study designed to investigate the prevalence of ATLL in this community suggested that the annual incidence in African Americans of Afro-Caribbean descent was around 3.2/100,000 person-years, with the prevalence in females being higher than that of males at a 3:1 ratio [39]. Additional studies investigated the prevalence of HTLV-1 infection and subsequent ATLL in immigrant populations in south Florida, suggesting a unique prevalence in those of Caribbean descent, particular those from Haiti, Jamaica, and the Bahamas [40]. ATLL in these US Afro-Caribbean patients often presents more aggressively than in Japanese patient populations, despite the advent of modern therapies [41,42].

## 3. Aspects of the Biology of HTLV-1 Infection

As a positive-strand RNA retrovirus, HTLV-1 is categorized into several subtypes, labeled A-G. The classifications of these subtypes depend on variations in the 3’ end long terminal repeat (LTR) sequence [43]. The frequency of each subtype correlates greatly with geographic location. For example, the HTLV-1C subtype is the most genetically distant from the others and predominates in Australia, where it infects up to 30% of the nation’s indigenous population [7].

All subtypes of HTLV-1 integrate their genome, through reverse transcription, into the host cell’s genomic DNA, therefore causing life-long infection. There are two primary mechanisms of viral propagation: the infectious and mitotic pathways. The infectious pathway involves the transmission of HTLV-1 from infected to uninfected cells. The mitotic pathway involves the expansion of HTLV-1-infected T-cells that results from the replication of an HTLV-1-infected T-cell into two HTLV-1-infected daughter T-cells.

Although HTLV-1 can infect dendritic cells, B-cells, and macrophages, the polyclonal expansion of CD4+ T-cells is the hallmark of infection [1]. The infectious pathway requires cell-to-cell contact through virological synapses, cellular conduits, biofilm-like extracellular viral assemblies, and extracellular vesicles [44,45,46]. The first two of these modalities resemble immunological synapses containing center, peripheral, and distal supramolecular activation complexes (SMACs) to ensure a secure conduit from one cell to another. HTLV-1 creates a similar controlled environment to pass from antigen-presenting dendritic cells to T-cells [47].

HTLV-1’s genome also includes the genes *gag*, *pro, pol,* and *env*, which encode structural proteins and functional enzymes that are conserved across all retroviruses [48]. Along with the accessory and regulatory proteins p8, p12, p13, and p30, the *Tax* and *HBZ* genes each play key roles in viral infection (see below) [49,50].

## 4. Mechanisms of Oncogenesis and Immune Dysregulation in HTLV-1 Infection

In addition to its transforming role in mature T-cells, a critical complication of ATLL malignancy secondary to HTLV-1 infection is profound immunosuppression, often leading to the development of opportunistic infections (OIs). Many of the same OIs are found in other immunocompromised patients, especially those with HIV/AIDS. These include *Pneumocystis jirovecii*, aspergillosis, candidiasis, and strongyloidiasis [51]. One of the primary mechanisms of immune suppression in ATLL occurs through excessive cytokine production, resulting in chronic inflammation, persistent immune stimulation, and the eventual exhaustion of T-cells and natural killer (NK) cells [52]. The phenomenon of immunological exhaustion is driven strongly by the viral *Tax* and *HBZ* genes by distinct yet synergistic mechanisms.

**Figure 1:**
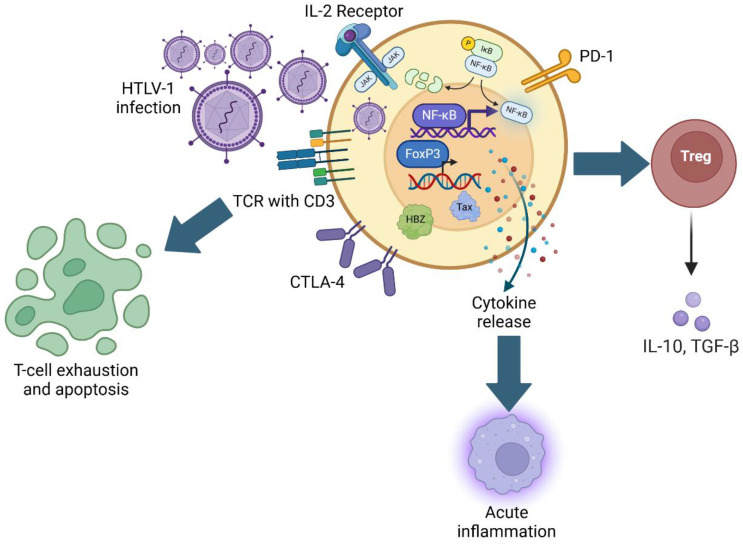
Mechanisms of HTLV-1-mediated immunosuppression. Schematic representation of HTLV-1 infection and subsequent upregulation of FoxP3 and NF-κB gene expression, modulation of T-cell activation markers, and relationship with acute inflammation, Treg function, and T-cell exhaustion.

As a highly immunogenic protein, Tax is expressed both transiently and in bursts, and persistent *Tax* expression has been shown to lead to the generation of Tax-specific cytotoxic T-lymphocytes (CTLs) that may play a role in limiting the viral infection of other T-cells. Periods of *Tax* expression result in a marked increase in nuclear factor kappa B (NF-κB) expression in HTLV-1-infected cells, stimulating the production of many immune-inflammatory cytokines [53,54,55]. In addition, NF-κB activation has been implicated in immune cell proliferation and the upregulation of anti-apoptotic gene products, further contributing to the transformation and aberrant survival of the immortalization of CD4^+^ T-cells (Figure 1) [56]. At the molecular level, Tax subverts the physiologic regulation of NF-κB, consistently associating with and activating the inducible IκB kinase (IKK), leading to the degradation of the endogenous inhibitor of NF-κB, IκBα, and resulting in sustained deregulated NF-κB expression [57,58]. The persistent increase in NF-κB activation consequently leads to the generation of the pro-inflammatory cytokines TNF-α, IL-2, and IL-6, which contribute to immune exhaustion [52]. In addition, Tax expression compounds the process of T-cell exhaustion via the excessive upregulation of IL-2 receptors (IL2RA) and HLA Class II proteins [59]. These changes exaggerate a state of chronic hyperstimulation that ultimately creates tolerogenic antigen-presenting cells (APCs), potentially leading to antigen-specific T-cell tolerance [59]. HTLV-1 also directly infects monocytes and macrophages. In this context, the p13 viral protein has been shown to influence macrophage polarization to interfere with the antiviral M1 macrophage phenotype and cause macrophages to increase chemokine levels to attract CD4+ lymphocytes [60]. In addition to promoting the anergy of antigen-presenting cells and contributing to T-cell exhaustion, the HTLV-1 infection of CD4^+^ lymphocytes leads to their functional developmental transition to immunosuppressive T regulatory cells (T_reg_). Although this process is driven predominantly by the effects of virally encoded HBZ, Tax expression also results in the release of IL-10, a potent immunosuppressive cytokine that suppresses T-cell proliferation and hampers the activity of macrophages [61].

Precisely pinpointing which of the several downstream effects of Tax expression led to the oncogenesis of ATLL has been difficult using in vivo syngeneic or transgenic mouse models [62]. In fact, across several experimental approaches, different tissue-specific expression of *Tax* has not been associated with the onset of an ATLL-like disease but rather a spectrum of other tumors, both epithelial and hematopoietic, including mesenchymal tumors emerging after Tax expression under the control of the CD3-ε promoter [63].

Unlike *Tax*, the *HBZ* gene is expressed continuously in HTLV-1-infected T-lymphocytes. The HBZ protein promotes the transcription of forkhead box protein 3 (FOXP3), the principal regulatory transcription factor that confers a T_reg_ phenotype to infected cells [64]. In addition, HBZ also causes an increase in the expression of CCR4, a chemokine receptor that is also associated with T_reg_ cells [65]. Beyond directing infected cells towards an immunosuppressive phenotype, HBZ has also been proposed to inhibit the expression or function of immune inhibitory receptors such as Programmed Cell Death-1 (PD-1) and T-Cell Immunoreceptor with Immunoglobulin and ITIM Domain (TIGIT), leading to constitutive, deregulated TCR activation [66]. Analogous to the mechanisms of the Tax protein, continuous TCR stimulation promotes the proliferation of infected T-cells while also eventually leading to exhaustion [66]. In current transgenic mouse models, HBZ expression under the control of the granzyme B promoter has yielded T-cell lymphoma and systemic inflammation, results consistent with the proposed oncogenic effects of HBZ in vivo [67].

Similar results were obtained in experiments using transformed ATLL cells [68]. Here, the abnormally upregulated expression of costimulatory markers (CD99, CD28, CD278) and activation markers (CD71, CD25, CD38), combined with the increased expression of PD-L1 and the immunosuppressive CD73 and CD39 surface proteins, concurs to drive Tax-specific CTLs towards anergy, effectively evading immune response [68].

Thus, the oncogenesis of ATLL is also believed to be driven by the expression of *Tax* and *HBZ* oncogenic factors. *Tax* serves to exploit a panoply of intracellular signaling and gene expression pathways as illustrated above while also contributing to clonal T-cell proliferation. As a highly immunogenic protein, *Tax* is transiently expressed to avoid eliciting the generation of Tax-specific CD8^+^ cytotoxic T-lymphocytes. Tax is responsible for activating T-cells and driving proliferation, particularly through its activation of the NF-κB pathway [62]. Tax expression is the highest during initial infection, but due to its strong immunogenic features, it becomes silent in most HTLV-1 infections [62,69]. However, while *Tax* is near undetectable in ATLL, the survival of leukemic cells continues to depend on Tax expression [70]. HBZ is responsible for driving FOXP3 expression in naïve T-cells as well as E2F1 and its targets to drive cell growth and T-cell differentiation [6,8,69]. Given this, it has been speculated that ATLL may originate from T_reg_ precursors and may be able to suppress T_helper_ lymphocytes and contribute to the opportunistic infections associated with ATLL [11,12,71].

## 5. HTLV-1-Related Diseases

### 5.1. Adult T-Cell Leukemia/Lymphoma

ATLL is an aggressive MTCN with substantial clinical heterogeneity. It demonstrates a mature T-cell phenotype (CD3^+^, CD4^+^, CD8^−^, and CD25^+^) with abnormal, multi-lobed nuclei (“flower cells”). It is a complication in about 3–5% of HTLV-1 cases [18]. Its distribution correlates with the worldwide distribution of HTLV-1, with ATLL representing 25% of MTCNs in Japan but only 1% in Europe [71]. It is a disease of adults, with a latency period of at least 20–30 years between initial infection with HTLV-1 and the development of ATLL [8,72].

ATLL is divided into four subtypes by the Shimoyama criteria: chronic, smoldering, acute, and lymphoma [73]. The acute and lymphoma subtypes represent aggressive disease, while the smoldering and chronic types represent more indolent disease. The clinical features of the disease are thus dependent on the subtype, but typical presenting features include lymphadenopathy, circulating abnormal lymphocytes with cerebriform nuclei, cutaneous lesions, hepatosplenomegaly, hypercalcemia, and infections. Other infections observed are similar to those associated with AIDS, including *Pneumocystis carinii*, aspergillosis, candidiasis, cytomegalovirus pneumonia, and *S. stercoralis*, as well as two malignancies: Kaposi’s sarcoma and Epstein–Barr virus (EBV)-associated B-cell lymphoma [74,75,76]. Prognosis is also dependent on subtype: aggressive ATLL confers a poor overall prognosis (median overall survival [mOS] 6–10 months), whereas indolent subtypes show an mOS around 2 years [77,78].

While observation is typically recommended for indolent disease, aggressive or symptomatic disease is treated with multiagent chemotherapy. Most trials for ATLL therapy are conducted in Japan by the Japanese Clinical Oncology Group (JCOG). The standard of care in Japan is sequential vincristine, cyclophosphamide, doxorubicin, prednisone–doxorubicin, ranimustine and prednisone, vindesine, etoposide, carboplatin, prednisone (VCAP-AMP-VECP) after the JCOG 9801 phase III trial showed a superior mOS compared to biweekly cyclophosphamide, vincristine, doxorubicin, prednisone (CHOP-14) [79]. The regimens used in the US include dose-adjusted etoposide, prednisolone, vincristine, cyclophosphamide, doxorubicin (DA-EPOCH) and hyper-fractionated cyclophosphamide, vincristine, doxorubicin, dexamethasone (hyper-CVAD) given the increase in toxicity seen with VCAP-AMP-VECP and the lack of vindesine and ranimustine availability in the US [80,81]. An alternative first-line therapy is the combination of zidovudine and interferon alpha, with or without combination chemotherapy. The mechanism of action of this combination has not been well elucidated, but current evidence suggests a possible antiviral, opposed to a direct cytotoxic, mechanism, as it has been shown to inhibit reverse transcriptase activity [82,83]. While no trials have been conducted comparing antivirals to chemotherapy, a meta-analysis of 238 patients demonstrated a first-line antiviral to have an mOS of 17 months vs. 10 months for chemotherapy (*p* = 0.004) [84]. When broken down by ATLL subtype, chronic and smoldering ATLL showed a 100% 5-year survival for antivirals against 42% with chemotherapy alone (*p* = 0.001) but showed significantly worse survival in the lymphoma subtype with a 5-year survival of 0% for antivirals against 18% for chemotherapy with or without maintenance antivirals (*p* = 0.009). For elderly patients, CHOP-14 was combined with mogamulizumab, a monoclonal antibody targeting CCR4, which was shown to have promising results in a phase II trial with an overall response rate (ORR) of 91.7% with a complete response rate (CR) of 64.6% [85].

Consolidation with allogeneic stem cell transplant remains a common treatment strategy for aggressive subtypes of ATLL, though there are unfortunately no prospective studies investigating its utility. A nationwide retrospective study of Japan in 2010 demonstrated a 3-year OS of 33% for transplant, but treatment-related mortality was as high as 43% [86]. This high treatment-related mortality has been speculated to be due to the immune suppression innate to ATLL in combination with the fact that it is a disease common in older adults. This high treatment-related mortality must be weighed against a 2-year survival of 15–20% for patients with aggressive ATLL without transplant [87].

Relapsed or primary refractory ATLL remains an unmet need. Treatment often consists of single-agent therapy. Agents that have been investigated include monoclonal antibodies (mogamulizumab, alemtuzumab, daclizumab), bortezomib, and lenalidomide, amongst others [88,89,90,91,92]. One novel approach for studies in ATLL is a therapeutic vaccine. One such vaccine involves autologous dendritic cells pulsed with the Tax protein, which showed promising results in a pilot trial of three patients [93]. Another vaccine involves a lentiviral vector targeted against viral Tax, HBZ, p12I, and p30II proteins [94].

### 5.2. HTLV-1-Associated Myelopathy/Tropical Spastic Paresis

HAM/TSP is a chronic and progressive spastic paresis. HAM/TSP incidence varies by region with estimates lower in Japanese patients (<1% lifetime risk) than Caribbean populations (~2%) while also showing increased risk in women compared to men [95,96]. The pathophysiology is believed to involve a bystander effect involving the CD8+ cytotoxic T-cell destruction of CD4+ HTLV-1-infected T-cells leading to the demyelination of the central nervous system, particularly of the spinal cord [97,98]. Common symptoms include weakness of the lower limbs with associated spasticity, paresthesias, urinary incontinence, and sexual dysfunction [98,99,100]. Diagnosis involves typical neurological symptoms with the demonstration of HTLV-1 infection—HTLV-1 provirus and antibodies can be isolated from the cerebrospinal fluid of patients [101].

High-quality evidence guiding treatment is currently limited, with standard-of-care agents including corticosteroids and interferon. The HAMLET-P trial was a placebo-controlled study looking at corticosteroids in the first line (rapid progressors were also randomized for 3-day methylprednisolone pulse vs. no steroid pulse) and found no statistically significant difference between oral prednisolone 0.5mg/kg with taper for their primary outcome of improvement in the disability score [102]. Interferon-α has been approved for use in Japan, but long-term use rates are only ~3%, and it has been associated with worsened outcomes—it is not approved for use in the US [103]. Other agents under investigation include raltegravir (viral integrase inhibitor) and mogamulizumab, which have shown decreased viral load in early-phase studies [104,105]. Ultimately, further studies and more high-quality evidence are required, and treatment options remain limited for this disease.

### 5.3. HTLV-1-Associated Uveitis

HTLV-1-associated uveitis (HU) is caused by HTLV-1 and is characterized by vision changes, blurry vision, hyperemia, ocular pain, and photophobia. In Japan, it is estimated at a prevalence of 112.2 per 100,000 persons infected with HTLV-1 with a predilection for females [106]. It is a diagnosis of exclusion, with patients demonstrating seropositivity for HTLV-1 and no other documented causes of uveitis demonstrated. Pathogenesis is not fully elucidated, but aqueous humor evaluation reveals CD4+ T-cells that demonstrate viral infection at rates higher compared to mononuclear cells in the blood, and PCR has demonstrated HTLV-1 in the aqueous humor of infected individuals [107,108]. The disease is thought to be driven by cytokine release (particularly IL-6 and TNF-α), rather than a clonal process, as T-cell receptor studies have demonstrated that the cells are polyclonal [108,109,110]. Given its inflammatory etiology, treatment involves the use of both local (e.g., intravitreal) and systemic steroids. Studies for specific treatments of HU are lacking and are an area of unmet need. In a retrospective study of 28 affected eyes treated with injected and/or systemic steroids, only two had a decrease in vision [111].

### 5.4. Infective Dermatitis

HTLV-1-associated infective dermatitis (HAID) is the predominant manifestation of HTLV-1 in children (average age of onset is around 2 years) in contrast to ATLL, HAM/TSP, and HU which affect adults. It is seen most frequently in the Caribbean, particularly Jamaica, and Brazil with few cases reported in other endemic areas such as Japan despite the virus’s higher prevalence [112,113,114]. It is associated with the vertical transmission of HTLV-1, particularly through breastfeeding. The disease manifests as a relapsing/remitting, chronic eczematous rash with superinfection with *Staphylococcus aureus* and/or group B hemolytic Streptococcus. Other key features include hypergammaglobulinemia, lymphadenopathy, lymphocytosis, and an elevated CD4/CD8 ratio [112]. The condition is hypothesized to be driven by immunosuppression by HTLV-1 infection. Interestingly, compared to control subjects with atopic dermatitis where lesional T-lymphocytes predominantly demonstrate the T_helper_ phenotype, HAID biopsies demonstrate CD8+ lymphocytes that are perforin-negative, indicating inactive CD8+ T-cells [115]. Treatment involves a prolonged course of antibiotics, classically with trimethoprim–sulfamethoxazole, and patients often respond well though relapses upon the discontinuation of antibiotics are common [113,116]. The condition generally goes into remission as affected children age, typically around age 15 [113].

### 5.5. Non-HTLV-1-Associated Illnesses

The above conditions are all causally associated with HTLV-1, but there is a growing body of literature suggesting that HTLV-1 infection can affect other disease processes. A recent meta-analysis including case–control, cohort, and cross-sectional studies from multiple countries including those in North America, South America, Central America, Asia, the Middle East, Africa, and Australia found a 56% increase in all-cause mortality with strong evidence [117]. The study also noted weaker evidence for an increase in non-ATLL malignancies (cervical, gastric, other lymphomas), certain infections (including Tuberculosis, genitourinary, and Strongyloidiasis), and inflammatory conditions (including fibromyalgia, dermatitis, rheumatoid arthritis, and eczema), but the authors point out that the increase in these conditions is not sufficient to explain the degree of all-cause mortality, implying that HTLV-1 is influencing survival in a way independent of increasing these pathologies. Notably, in a retrospective study in Peru, patients with cervical cancer, breast cancer, and non-ATLL, aggressive non-Hodgkin lymphomas were shown to have lower 5-year OS rates (47%, 56%, and 30%, respectively) compared to historical outcomes (60%, 70%, and 40–50%, respectively) [118]. While there are many confounders in these retrospective studies, the established inflammatory milieu created by HTLV-1 infection has also been implicated in cardiovascular illness, and HTLV-1 infection has been shown to be an independent risk factor in the development of arteriosclerosis [119]. Further research is needed to better understand the implications of HTLV-1 in other disease states and the underpinnings of how it influences outcomes.

## 6. Conclusions and Future Directions

Since the initial isolation of HTLV-1 in 1980, enormous progress has been made in characterizing this oncogenic retrovirus. The discovery of cell-to-cell contact for infection, the characteristic proviral integration into the host genome, and the roles of the virally encoded Tax and HBZ proteins have come into better focus, shedding light on how HTLV-1 survives and replicates in vivo and how it can drive human diseases with currently dismal prognoses. Several questions remain unanswered, however. Research into the development of ATLL has highlighted risk factors, but no true causal mechanism relating these risk factors to the development of ATLL has been established.

Beyond risk factors, the causes of the decades-long delay in the development of ATLL and the most common triggers for oncogenesis are unknown and remain difficult to find, given the rarity and underreporting of HTLV-1-associated diseases. Future directions of HTLV-1 research should predominantly focus on the improvement in epidemiological data, leading to the greater awareness and education of at-risk populations. In this context, strategies to develop reliable in vivo models of HTLV-1-driven human diseases to facilitate the development of novel therapeutics as well as preventative or even therapeutic vaccines are key priorities. Similarly, successful examples of improved community awareness and public health policies to limit viral transmission exist that could be more broadly reproduced in emerging at-risk and vulnerable populations.

The progress of collecting and processing epidemiological data of HTLV-1 infection in endemic areas has improved, but large gaps remain. Although widespread, representative epidemiological studies have yet to be conducted on a consistent basis, the multifactorial nature of HTLV-1 spread as a sexually transmitted disease, a blood-borne illness, and through vertical transmission has become clearer. Difficulties in identifying and confirming HTLV-1-positive individuals presents the most significant, daunting challenge. Due to the dormant nature of infection, with most seropositive individuals remaining asymptomatic for life, along with resource constraints in many endemic areas, it remains nearly impossible to accurately estimate the prevalence of HTLV-1 in the general population.

While HTLV-1-associated human diseases are rare in the US, the estimated unmet medical need and public health challenge they pose is substantial. In more than 80% of cases, ATLL is a very aggressive malignancy with acute clinical presentation, multiorgan failure, and severe metabolic abnormalities. Most patients are urgently admitted through the Emergency Department, requiring intensive supportive care and the immediate initiation of chemotherapy. While specific hospital mortality rates for newly diagnosed aggressive ATLL are not readily available, the median survival is less than 6 months, and the 4-year survival rate is about 10% [22].

Because in the US HTLV-1 disproportionally affects communities of immigrants, with language barriers, low socioeconomic status, and limited access to healthcare, ATLL is rarely diagnosed before it produces life-threatening complications. Even in the minority (~20%) of cases with a more indolent presentation, the disease eventually accelerates and transitions to a more aggressive course. This unfavorable clinical landscape highlights the need for public health strategies to limit viral spread, improve community awareness, and curtail the expansion of asymptomatic carriers, but these have been unevenly applied to this point [23].

**Figure 2:**
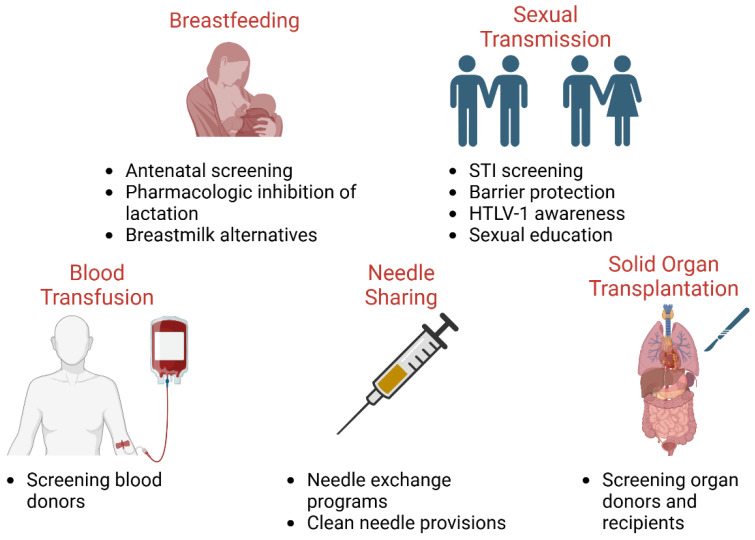
Public health mitigation strategies to control HTLV-1 spread. Public policies and protocols to limit the spread in underserved populations include reducing breastfeeding in HTLV-1-positive mothers, safe sex practices and disease testing, blood and organ donor screening, and harm-reducing needle exchange programs.

These strategies, therefore, should target the prevention and early diagnosis of ATLL, ideally through accurate risk assessment and the quantification of risk. Given that higher proviral loads are associated with greater transmission risks as compared to individuals with lower proviral loads, it is consequential that these high-risk individuals are identified early on [120]. Once high-risk individuals are identified, the next key steps would be to mitigate the avenues by which HTLV-1 transmission most often occurs, namely through mother-to-child transmission (Figure 2). Although for an HTLV-1-infected mother, refraining from breastfeeding can prevent 87% of early-life infection, there may be instances in which exclusive formula feeding is not feasible, affordable, or sustainable—in such cases, a systemic review has found that short-term breastfeeding for up to only 3 months of a child’s life can be considered [121]. Other notable avenues of transmission include sexual transmission and needle sharing with intravenous drug use [122]. These preventative measures would be a greater undertaking, requiring major efforts to increase access to specialized care for accelerated diagnosis, treatment, and disease management through more efficient laboratory testing facilities and community education. This should be especially implemented in rural areas, with a higher prevalence of HTLV-1, where geographic inaccessibility to healthcare is of serious concern [38].

As we illustrated, HTLV-1 disproportionately burdens vulnerable populations of low socioeconomic status, a trend that has emerged in several endemic regions, such as Africa and Brazil. While HTLV-1-associated diseases are rare, especially outside of endemic areas, globalization and migration trends have led to the emergence of HTLV-1-related diseases, as seen in the United States. HTLV-1 prevalence and new viral transmission in nascent and growing immigrant communities in the United States can be expected to increase. A number of public health measures implemented in some endemic areas can be used to combat the spread of HTLV-1 in high-risk populations in non-endemic regions. These include community awareness, freely accessible and encouraged testing, education on the risks of breastfeeding, harm reduction programs for needle use, and safe sexual practices. 

## Data Availability

No new data was created for this manuscript.

## References

[1] Poiesz B.J., Ruscetti F.W., Gazdar A.F., Bunn P.A., Minna J.D., Gallo R.C. (1980). Detection and isolation of type C retrovirus particles from fresh and cultured lymphocytes of a patient with cutaneous T-cell lymphoma. Proc. Natl. Acad. Sci. USA.

[2] Popovic M., Reitz M.S., Sarngadharan M.G., Robert-Guroff M., Kalyanaraman V.S., Nakao Y., Miyoshi I., Minowada J., Yoshida M., Ito Y. (1982). The virus of Japanese adult T-cell leukaemia is a member of the human T-cell leukaemia virus group. Nature.

[3] Hinuma Y., Nagata K., Hanaoka M., Nakai M., Matsumoto T., Kinoshita K.I., Shirakawa S., Miyoshi I. (1981). Adult T-cell leukemia: Antigen in an ATL cell line and detection of antibodies to the antigen in human sera. Proc. Natl. Acad. Sci. USA.

[4] Osame M., Usuku K., Izumo S., Ijichi N., Amitani H., Igata A., Matsumoto M., Tara M. (1986). HTLV-I associated myelopathy, a new clinical entity. Lancet.

[5] Mochizuki M., Watanabe T., Yamaguchi K., Tajima K., Yoshimura K., Nakashima S., Shirao M., Araki S., Miyata N., Mori S. (1992). Uveitis associated with human T lymphotropic virus type I: Seroepidemiologic, clinical, and virologic studies. J. Infect. Dis..

[6] Cook L.B., Taylor G.P. (2014). HTLV-1 and HTLV-2 prevalence in the United States. J. Infect. Dis..

[7] Gessain A., Cassar O. (2012). Epidemiological Aspects and World Distribution of HTLV-1 Infection. Front. Microbiol..

[8] Proietti F.A., Carneiro-Proietti A.B.F., Catalan-Soares B.C., Murphy E.L. (2005). Global epidemiology of HTLV-I infection and associated diseases. Oncogene.

[9] Edlich R.F., Hill L.G., Williams F.M. (2003). Global epidemic of human T-cell lymphotrophic virus type-I (HTLV-I): An update. J. Long Term Eff. Med. Implant..

[10] Shah U.A., Shah N., Qiao B., Acuna-Villaorduna A., Pradhan K., Herrera D.A., Sica R.A., Shastri A., Mantzaris I., Derman O. (2020). Epidemiology and survival trend of adult T-cell leukemia/lymphoma in the United States. Cancer.

[11] De la Fuente L., Toro C., Soriano V., Brugal M.T., Vallejo F., Barrio G., Jiménez V., Silva T., Project Itínere Working Group (2006). HTLV infection among young injection and non-injection heroin users in Spain: Prevalence and correlates. J. Clin. Virol..

[12] Feigal E., Murphy E., Vranizan K., Bacchetti P., Chaisson R., Drummond J.E., Blattner W., McGrath M., Greenspan J., Moss A. (1991). Human T cell lymphotropic virus types I and II in intravenous drug users in San Francisco: Risk factors associated with seropositivity. J. Infect. Dis..

[13] Itabashi K., Miyazawa T., Uchimaru K. (2023). How Can We Prevent Mother-to-Child Transmission of HTLV-1?. Int. J. Mol. Sci..

[14] Marino-Merlo F., Grelli S., Mastino A., Lai M., Ferrari P., Nicolini A., Pistello M., Macchi B. (2023). Human T-Cell Leukemia Virus Type 1 Oncogenesis between Active Expression and Latency: A Possible Source for the Development of Therapeutic Targets. Int. J. Mol. Sci..

[15] Sagara Y., Nakamura H., Satake M., Watanabe T., Hamaguchi I. (2022). Increasing horizontal transmission of human T-cell leukemia virus type 1 in adolescents and young adults in Japan. J. Clin. Virol..

[16] Cook L.B.M., Melamed A., Demontis M.A., Laydon D.J., Fox J.M., Tosswill J.H.C., de Freitas D., Price A.D., Medcalf J.F., Martin F. (2016). Rapid dissemination of human T-lymphotropic virus type 1 during primary infection in transplant recipients. Retrovirology.

[17] Iwanaga M. (2020). Epidemiology of HTLV-1 Infection and ATL in Japan: An Update. Front. Microbiol..

[18] Iwanaga M., Watanabe T., Yamaguchi K. (2012). Adult T-cell leukemia: A review of epidemiological evidence. Front. Microbiol..

[19] Yoshizumi T., Shirabe K., Ikegami T., Kayashima H., Yamashita N., Morita K., Masuda T., Hashimoto N., Taketomi A., Soejima Y. (2012). Impact of human T cell leukemia virus type 1 in living donor liver transplantation. Am. J. Transplant..

[20] Umekita K., Okayama A. (2020). HTLV-1 Infection and Rheumatic Diseases. Front. Microbiol..

[21] Satoh M., Toma H., Sugahara K., Etoh K.-I., Shiroma Y., Kiyuna S., Takara M., Matsuoka M., Yamaguchi K., Nakada K. (2002). Involvement of IL-2/IL-2R system activation by parasite antigen in polyclonal expansion of CD4^+^25^+^ HTLV-1-infected T-cells in human carriers of both HTLV-1 and S. stercoralis. Oncogene.

[22] Valcarcel B., Idrobo H., Pavlovsky A., Miranda E.C., Beltran B., Paredes S., Enriquez-Vera D., Vasquez J.F., Roche C., Valvert F. (2025). Prevalence and survival outcomes of adult T-cell leukemia/lymphoma in Latin America: A multicenter cohort study and recommendations to improve diagnosis and outcomes. Cancer Epidemiol..

[23] Rosadas C., Menezes M.L.B., Galvão-Castro B., Assone T., Miranda A.E., Aragón M.G., Caterino-De-Araujo A., Taylor G.P., Ishak R. (2021). Blocking HTLV-1/2 silent transmission in Brazil: Current public health policies and proposal for additional strategies. PLoS Negl. Trop. Dis..

[24] Ichikawa T., Taura N., Miyaaki H., Matsuzaki T., Ohtani M., Eguchi S., Takatsuki M., Soyama A., Hidaka M., Okudaira S. (2012). Human T-cell leukemia virus type 1 infection worsens prognosis of hepatitis C virus-related living donor liver transplantation. Transpl. Int..

[25] Taylor G.P. (2018). Human T-lymphotropic virus type 1 infection and solid organ transplantation. Rev. Med. Virol..

[26] Yamauchi J., Yamano Y., Yuzawa K. (2019). Risk of Human T-Cell Leukemia Virus Type 1 Infection in Kidney Transplantation. N. Engl. J. Med..

[27] Oliveira-Filho A.B., Araújo A.P.S., Souza A.P.C., Gomes C.M., Silva-Oliveira G.C., Martins L.C., Fischer B., Machado L.F.A., Vallinoto A.C.R., Ishak R. (2019). Human T-lymphotropic virus 1 and 2 among people who used illicit drugs in the state of Pará, northern Brazil. Sci. Rep..

[28] Ishak R., Harrington W.J., Azevedo V.N., Eiraku N., Ishak M.O., Guerreiro J.F., Santos S.B., Kubo T., Monken C., Alexander S. (1995). Identification of human T cell lymphotropic virus type IIa infection in the Kayapo, an indigenous population of Brazil. AIDS Res. Hum. Retroviruses.

[29] Paiva A.M., Assone T., Haziot M.E.J., Smid J., Fonseca L.A.M., Luiz O.D.C., de Oliveira A.C.P., Casseb J. (2018). Risk factors associated with HTLV-1 vertical transmission in Brazil: Longer breastfeeding, higher maternal proviral load and previous HTLV-1-infected offspring. Sci. Rep..

[30] Rosadas C., Taylor G.P. (2019). Mother-to-Child HTLV-1 Transmission: Unmet Research Needs. Front. Microbiol..

[31] Martin F., Tagaya Y., Gallo R. (2018). Time to eradicate HTLV-1: An open letter to WHO. Lancet.

[32] Mauclère P., Le Hesran J., Mahieux R., Salla R., Mfoupouendoun J., Abada E.T., Millan J., de Thé G., Gessain A. (1997). Demographic, ethnic, and geographic differences between human T cell lymphotropic virus (HTLV) type I-seropositive carriers and persons with HTLV-I Gag-indeterminate Western blots in Central Africa. J. Infect. Dis..

[33] Verdier M., Denis F., Sangaré A., Barin F., Gershy-Damet G., Rey J.-L., Soro B., Léonard G., Mounier M., Hugon J. (1989). Prevalence of antibody to human T cell leukemia virus type 1 (HTLV-1) in populations of Ivory Coast, West Africa. J. Infect. Dis..

[34] Delaporte E., Peeters M., Bardy J.L., Ville Y., Placca L., Bedjabaga I., Larouzé B., Piot P. (1993). Blood transfusion as a major risk factor for HTLV-I infection among hospitalized children in Gabon (Equatorial Africa). J. Acquir. Immune Defic. Syndr..

[35] Del Mistro A., Chotard J., Hall A.J., Fortuin M., Whittle H., DE Rossi A., Chieco-Bianchi L. (1994). HTLV-I/II seroprevalence in The Gambia: A study of mother-child pairs. AIDS Res. Hum. Retroviruses.

[36] Van Tienen C., McConkey S.J., De Silva T.I., Cotten M., Kaye S., Sarge-Njie R., da Costa C., Gonçalves N., Parker J., Vincent T. (2012). Maternal proviral load and vertical transmission of human T cell lymphotropic virus type 1 in Guinea-Bissau. AIDS Res. Hum. Retroviruses.

[37] Cèsaire R., Bera O., Maier H., Lezin A., Martial J., Ouka M., Kerob-Bauchet B., Amar A.O., Vernant J. (1999). Seroindeterminate patterns and seroconversions to human T-lymphotropic virus type I positivity in blood donors from Martinique, French West Indies. Transfusion.

[38] Gessain A., Ramassamy J.-L., Afonso P.V., Cassar O. (2023). Geographic distribution, clinical epidemiology and genetic diversity of the human oncogenic retrovirus HTLV-1 in Africa, the world’s largest endemic area. Front. Immunol..

[39] Levine P.H., Dosik H., Joseph E.M., Felton S., Bertoni M.A., Cervantes J., Moulana V., Miotti A.B., Goberdhan L.J., Lee S.L. (1999). A study of adult T-cell leukemia/lymphoma incidence in central Brooklyn. Int. J. Cancer.

[40] Harrington W.J., Miller G.A., Kemper R.R., Byrne G.E., Whitcomb C.C., Rabin M. (1991). HTLV-I-associated leukemia/lymphoma in south Florida. J. Acquir. Immune Defic. Syndr..

[41] Malpica L., Pimentel A., Reis I.M., Gotuzzo E., Lekakis L., Komanduri K., Harrington T., Barber G.N., Ramos J.C. (2018). Epidemiology, clinical features, and outcome of HTLV-1-related ATLL in an area of prevalence in the United States. Blood Adv..

[42] Zell M., Assal A., Derman O., Kornblum N., Battini R., Wang Y., Narasimhulu D.M., Mantzaris I., Shastri A., Verma A. (2016). Adult T-cell leukemia/lymphoma in the Caribbean cohort is a distinct clinical entity with dismal response to conventional chemotherapy. Oncotarget.

[43] Fujisawa J., Seiki M., Kiyokawa T., Yoshida M. (1985). Functional activation of the long terminal repeat of human T-cell leukemia virus type I by a trans-acting factor. Proc. Natl. Acad. Sci. USA.

[44] Pinto D.O., Al Sharif S., Mensah G., Cowen M., Khatkar P., Erickson J., Branscome H., Lattanze T., DeMarino C., Alem F. (2021). Extracellular vesicles from HTLV-1 infected cells modulate target cells and viral spread. Retrovirology.

[45] Pise-Masison C.A., Franchini G. (2022). Hijacking Host Immunity by the Human T-Cell Leukemia Virus Type-1: Implications for Therapeutic and Preventive Vaccines. Viruses.

[46] Van Prooyen N., Gold H., Andresen V., Schwartz O., Jones K., Ruscetti F., Lockett S., Gudla P., Venzon D., Franchini G. (2010). Human T-cell leukemia virus type 1 p8 protein increases cellular conduits and virus transmission. Proc. Natl. Acad. Sci. USA.

[47] Mulherkar T.H., Gómez D.J., Sandel G., Jain P. (2022). Co-Infection and Cancer: Host-Pathogen Interaction between Dendritic Cells and HIV-1, HTLV-1, and Other Oncogenic Viruses. Viruses.

[48] Boxus M., Willems L. (2009). Mechanisms of HTLV-1 persistence and transformation. Br. J. Cancer.

[49] Giam C.Z., Jeang K.T. (2007). HTLV-1 Tax and adult T-cell leukemia. Front. Biosci..

[50] Yasunaga J., Matsuoka M. (2011). Molecular mechanisms of HTLV-1 infection and pathogenesis. Int. J. Hematol..

[51] Maeda T., Babazono A., Nishi T., Yasui M., Matsuda S., Fushimi K., Fujimori K. (2015). The Impact of Opportunistic Infections on Clinical Outcome and Healthcare Resource Uses for Adult T Cell Leukaemia. PLoS ONE.

[52] Futsch N., Prates G., Mahieux R., Casseb J., Dutartre H. (2018). Cytokine Networks Dysregulation during HTLV-1 Infection and Associated Diseases. Viruses.

[53] El Hajj H., Bazarbachi A. (2022). Interplay between innate immunity and the viral oncoproteins Tax and HBZ in the pathogenesis and therapeutic response of HTLV-1 associated adult T cell leukemia. Front. Immunol..

[54] Kannagi M., Harashima N., Kurihara K., Ohashi T., Utsunomiya A., Tanosaki R., Masuda M., Tomonaga M., Okamura J. (2005). Tumor immunity against adult T-cell leukemia. Cancer Sci..

[55] Mohanty S., Harhaj E.W. (2020). Mechanisms of Oncogenesis by HTLV-1 Tax. Pathogens.

[56] Ernzen K.J., Panfil A.R. (2022). Regulation of HTLV-1 transformation. Biosci. Rep..

[57] Portis T., Harding J.C., Ratner L. (2001). The contribution of NF-kappa B activity to spontaneous proliferation and resistance to apoptosis in human T-cell leukemia virus type 1 Tax-induced tumors. Blood.

[58] Sun S.C., Ballard D.W. (1999). Persistent activation of NF-kappaB by the tax transforming protein of HTLV-1: Hijacking cellular IkappaB kinases. Oncogene.

[59] Tan B.J., Sugata K., Reda O., Matsuo M., Uchiyama K., Miyazato P., Hahaut V., Yamagishi M., Uchimaru K., Suzuki Y. (2021). HTLV-1 infection promotes excessive T cell activation and transformation into adult T cell leukemia/lymphoma. J. Clin. Investig..

[60] Moles R., Omsland M., Pise-Masison C.A., Subleski J.J., McVicar D.W., Sarkis S., Gutowska A., Schifanella L., Doster M., Washington-Parks R. (2025). HTLV-1 p13 Protein Hijacks Macrophage Polarization and Promotes T-Cell Recruitment. Viruses.

[61] Takeuchi M., Miyoshi H., Ohshima K. (2021). Tumor microenvironment of adult T-cell leukemia/lymphoma. J. Clin. Exp. Hematop..

[62] Nakajima S., Okuma K. (2023). Mouse Models for HTLV-1 Infection and Adult T Cell Leukemia. Int. J. Mol. Sci..

[63] Hasegawa H., Sawa H., Lewis M.J., Orba Y., Sheehy N., Yamamoto Y., Ichinohe T., Tsunetsugu-Yokota Y., Katano H., Takahashi H. (2006). Thymus-derived leukemia-lymphoma in mice transgenic for the Tax gene of human T-lymphotropic virus type I. Nat. Med..

[64] Madureira M.W., Queiroz M.A.F., Lima S.S., Pereira L.M., da Costa C.A., de Sousa M.S., Feitosa R.N., Monteiro J.C., Ishak R., Vallinoto A.C. (2023). The FOXP3-924 A/G Single Nucleotide Polymorphism May Be Associated with Predictive Factors for Human T Lymphotropic Virus 1 Associated Myelopathy. Viral. Immunol..

[65] Bangham C.R.M. (2023). HTLV-1 persistence and the oncogenesis of adult T-cell leukemia/lymphoma. Blood.

[66] Kataoka K., Nagata Y., Kitanaka A., Shiraishi Y., Shimamura T., Yasunaga J., Totoki Y., Chiba K., Sato-Otsubo A., Nagae G. (2015). Integrated molecular analysis of adult T cell leukemia/lymphoma. Nat. Genet..

[67] Satou Y., Yasunaga J.-I., Zhao T., Yoshida M., Miyazato P., Takai K., Shimizu K., Ohshima K., Green P.L., Ohkura N. (2011). HTLV-1 bZIP factor induces T-cell lymphoma and systemic inflammation in vivo. PLoS Pathog..

[68] Koya J., Saito Y., Kameda T., Kogure Y., Yuasa M., Nagasaki J., McClure M.B., Shingaki S., Tabata M., Tahira Y. (2021). Single-Cell Analysis of the Multicellular Ecosystem in Viral Carcinogenesis by HTLV-1. Blood Cancer Discov..

[69] Williams A.E., Fang C.T., Slamon D.J., Poiesz B.J., Sandler S.G., Darr W.F., Shulman G., McGowan E.I., Douglas D.K., Bowman R.J. (1988). Seroprevalence and epidemiological correlates of HTLV-I infection in U.S. blood donors. Science.

[70] Hleihel R., Skayneh H., de Thé H., Hermine O., Bazarbachi A. (2023). Primary cells from patients with adult T cell leukemia/lymphoma depend on HTLV-1 Tax expression for NF-kappaB activation and survival. Blood Cancer J..

[71] Vose J., Armitage J., Weisenburger D. (2008). International peripheral T-cell and natural killer/T-cell lymphoma study: Pathology findings and clinical outcomes. J. Clin. Oncol..

[72] GonçaLves D.U., Proietti F.A., Ribas J.G.R., Araújo M.G., Pinheiro S.R., Guedes A.C., Carneiro-Proietti A.B.F. (2010). Epidemiology, treatment, and prevention of human T-cell leukemia virus type 1-associated diseases. Clin. Microbiol. Rev..

[73] Shimoyama M. (1991). Diagnostic criteria and classification of clinical subtypes of adult T-cell leukaemia-lymphoma. A report from the Lymphoma Study Group (1984–87). Br. J. Haematol..

[74] White J.D., Zaknoen S.L., Kasten-Sportès C., Top L.E., Navarro-Roman L., Nelson D.L., Waldmann T.A. (1995). Infectious complications and immunodeficiency in patients with human T-cell lymphotropic virus I-associated adult T-cell leukemia/lymphoma. Cancer.

[75] Greenberg S.J., Jaffe E.S., Ehrlich G.D., Korman N.J., Poiesz B.J., Waldmann T.A. (1990). Kaposi’s sarcoma in human T-cell leukemia virus type I-associated adult T-cell leukemia. Blood.

[76] Kamachi K., Shindo T., Miyahara M., Kitaura K., Akashi M., Shin T., Suzuki R., Oshima K., Kimura S. (2019). Epstein-Barr virus-related diffuse large B-cell lymphoma in mogamulizumab-treated adult T-cell leukemia with incomplete T-cell reconstitution. Int. J. Hematol..

[77] Phillips A.A., Shapira I., Willim R.D., Sanmugarajah J., Solomon W.B., Horwitz S.M., Savage D.G., Bhagat G., Soff G., Zain J.M. (2010). A critical analysis of prognostic factors in North American patients with human T-cell lymphotropic virus type-1-associated adult T-cell leukemia/lymphoma: A multicenter clinicopathologic experience and new prognostic score. Cancer.

[78] Katsuya H., Ishitsuka K., Utsunomiya A., Hanada S., Eto T., Moriuchi Y., Saburi Y., Miyahara M., Sueoka E., Uike N. (2015). Treatment and survival among 1594 patients with ATL. Blood.

[79] Tsukasaki K., Utsunomiya A., Fukuda H., Shibata T., Fukushima T., Takatsuka Y., Ikeda S., Masuda M., Nagoshi H., Ueda R. (2007). VCAP-AMP-VECP compared with biweekly CHOP for adult T-cell leukemia-lymphoma: Japan Clinical Oncology Group Study JCOG9801. J. Clin. Oncol..

[80] Ratner L., Rauch D., Abel H., Caruso B., Noy A., Barta S.K., Parekh S., Ramos J.C., Ambinder R., Phillips A. (2016). Dose-adjusted EPOCH chemotherapy with bortezomib and raltegravir for human T-cell leukemia virus-associated adult T-cell leukemia lymphoma. Blood Cancer J..

[81] Alduaij A., Butera J.N., Treaba D., Castillo J. (2010). Complete remission in two cases of adult T-cell leukemia/lymphoma treated with hyper-CVAD: A case report and review of the literature. Clin. Lymphoma Myeloma Leuk..

[82] Macchi B., Balestrieri E., Frezza C., Grelli S., Valletta E., Marçais A., Marino-Merlo F., Turpin J., Bangham C.R., Hermine O. (2017). Quantification of HTLV-1 reverse transcriptase activity in ATL patients treated with zidovudine and interferon-alpha. Blood Adv..

[83] Bazarbachi A., Nasr R., El-Sabban M., Mahé A., Mahieux R., Gessain A., Darwiche N., Dbaibo G., Kersual J., Zermati Y. (2000). Evidence against a direct cytotoxic effect of alpha interferon and zidovudine in HTLV-I associated adult T cell leukemia/lymphoma. Leukemia.

[84] Bazarbachi A., Plumelle Y., Ramos J.C., Tortevoye P., Otrock Z., Taylor G., Gessain A., Harrington W., Panelatti G., Hermine O. (2010). Meta-analysis on the use of zidovudine and interferon-alfa in adult T-cell leukemia/lymphoma showing improved survival in the leukemic subtypes. J. Clin. Oncol..

[85] Yoshimitsu M., Choi I., Kusumoto S., Shimokawa M., Utsunomiya A., Suehiro Y., Hidaka T., Nosaka K., Sasaki H., Rai S. (2025). A phase 2 Trial of CHOP with Anti-CCR4 Antibody Mogamulizumab for older Patients with Adult T-Cell Leukemia/Lymphoma. Blood.

[86] Hishizawa M., Kanda J., Utsunomiya A., Taniguchi S., Eto T., Moriuchi Y., Tanosaki R., Kawano F., Miyazaki Y., Masuda M. (2010). Transplantation of allogeneic hematopoietic stem cells for adult T-cell leukemia: A nationwide retrospective study. Blood.

[87] Katsuya H., Yamanaka T., Ishitsuka K., Utsunomiya A., Sasaki H., Hanada S., Eto T., Moriuchi Y., Saburi Y., Miyahara M. (2012). Prognostic index for acute- and lymphoma-type adult T-cell leukemia/lymphoma. J. Clin. Oncol..

[88] Ishida T., Joh T., Uike N., Yamamoto K., Utsunomiya A., Yoshida S., Saburi Y., Miyamoto T., Takemoto S., Suzushima H. (2012). Defucosylated anti-CCR4 monoclonal antibody (KW-0761) for relapsed adult T-cell leukemia-lymphoma: A multicenter phase II study. J. Clin. Oncol..

[89] Sharma K., Janik J.E., O’MAhony D., Stewart D., Pittaluga S., Stetler-Stevenson M., Jaffe E.S., Raffeld M., Fleisher T.A., Lee C.C. (2017). Phase II Study of Alemtuzumab (CAMPATH-1) in Patients with HTLV-1-Associated Adult T-cell Leukemia/lymphoma. Clin. Cancer Res..

[90] Berkowitz J.L., Janik J.E., Stewart D.M., Jaffe E.S., Stetler-Stevenson M., Shih J.H., Fleisher T.A., Turner M., Urquhart N.E., Wharfe G.H. (2014). Safety, efficacy, and pharmacokinetics/pharmacodynamics of daclizumab (anti-CD25) in patients with adult T-cell leukemia/lymphoma. Clin. Immunol..

[91] Ishitsuka K., Utsunomiya A., Katsuya H., Takeuchi S., Takatsuka Y., Hidaka M., Sakai T., Yoshimitsu M., Ishida T., Tamura K. (2015). A phase II study of bortezomib in patients with relapsed or refractory aggressive adult T-cell leukemia/lymphoma. Cancer Sci..

[92] Ogura M., Imaizumi Y., Uike N., Asou N., Utsunomiya A., Uchida T., Aoki T., Tsukasaki K., Taguchi J., Choi I. (2016). Lenalidomide in relapsed adult T-cell leukaemia-lymphoma or peripheral T-cell lymphoma (ATLL-001): A phase 1, multicentre, dose-escalation study. Lancet Haematol..

[93] Suehiro Y., Hasegawa A., Iino T., Sasada A., Watanabe N., Matsuoka M., Takamori A., Tanosaki R., Utsunomiya A., Choi I. (2015). Clinical outcomes of a novel therapeutic vaccine with Tax peptide-pulsed dendritic cells for adult T cell leukaemia/lymphoma in a pilot study. Br. J. Haematol..

[94] Revaud D., Bejanariu A., Loussaief L., Sarry E., Zemmar A., Deplaine G., Coman T., Rossignol J., Hermine O., Bauche C. (2015). Development of an Anti-HTLV-1 Vaccine for the Treatment of Adult T-Cell Leukemia/Lymphoma. Blood.

[95] Kaplan J.E., Osame M., Kubota H., Igata A., Nishitani H., Maeda Y., Khabbaz R., Janssen R. (1990). The risk of development of HTLV-I-associated myelopathy/tropical spastic paraparesis among persons infected with HTLV-I. J. Acquir. Immune Defic. Syndr..

[96] Maloney E.M., Cleghorn F.R., Morgan O.S.C., Rodgers-Johnson P., Cranston B., Jack N., Blattner W.A., Bartholomew C., Manns A. (1998). Incidence of HTLV-I-associated myelopathy/tropical spastic paraparesis (HAM/TSP) in Jamaica and Trinidad. J. Acquir. Immune Defic. Syndr. Hum. Retrovirol..

[97] Nakamura T. (2023). HAM/TSP Pathogenesis: The Transmigration Activity of HTLV-1-Infected T Cells into Tissues. Pathogens.

[98] Yamano Y., Sato T. (2012). Clinical pathophysiology of human T-lymphotropic virus-type 1-associated myelopathy/tropical spastic paraparesis. Front. Microbiol..

[99] Orland J.R., Engstrom J., Fridey J., Sacher R.A., Smith J.W., Nass C., Garratty G., Newman B., Smith D., Wang B. (2003). Prevalence and clinical features of HTLV neurologic disease in the HTLV Outcomes Study. Neurology.

[100] De Castro-Costa C.M., Araújo A.Q., Barreto M.M., Takayanagui O.M., Sohler M.P., Da Silva E.L.M., De Paula S.M.B., Ishak R., Ribas J.G.R., Rovirosa L.C. (2006). Proposal for diagnostic criteria of tropical spastic paraparesis/HTLV-I-associated myelopathy (TSP/HAM). AIDS Res. Hum. Retroviruses.

[101] Lezin A., Olindo S., Olière S., Varrin-Doyer M., Marlin R., Cabre P., Smadja D., Cesaire R. (2005). Human T lymphotropic virus type I (HTLV-I) proviral load in cerebrospinal fluid: A new criterion for the diagnosis of HTLV-I-associated myelopathy/tropical spastic paraparesis?. J. Infect. Dis..

[102] Yamauchi J., Tanabe K., Sato T., Nakagawa M., Matsuura E., Tsuboi Y., Tamaki K., Sakima H., Ishihara S., Ohta Y. (2022). Efficacy of Corticosteroid Therapy for HTLV-1-Associated Myelopathy: A Randomized Controlled Trial (HAMLET-P). Viruses.

[103] Tsutsumi S., Sato T., Yagishita N., Yamauchi J., Araya N., Hasegawa D., Nagasaka M., Coler-Reilly A.L.G., Inoue E., Takata A. (2019). Real-world clinical course of HTLV-1-associated myelopathy/tropical spastic paraparesis (HAM/TSP) in Japan. Orphanet J. Rare Dis..

[104] Enose-Akahata Y., Billioux B.J., Azodi S., Dwyer J., Vellucci A., Ngouth N., Nozuma S., Massoud R., Cortese I., Ohayon J. (2021). Clinical trial of raltegravir, an integrase inhibitor, in HAM/TSP. Ann. Clin. Transl. Neurol..

[105] Sato T., Coler-Reilly A.L.G., Yagishita N., Araya N., Inoue E., Furuta R., Watanabe T., Uchimaru K., Matsuoka M., Matsumoto N. (2018). Mogamulizumab (Anti-CCR4) in HTLV-1-Associated Myelopathy. N. Engl. J. Med..

[106] Ikeda E., Ono A., Hikita N., Arima K., Mochizuki M., Yamaguchi K., Tajima K., Kiyokawa H. (1998). Estimated prevalence rate of HTLV-I uveitis in Chikugo. Nippon Ganka Gakkai Zasshi.

[107] Ono A., Mochizuki M., Yamaguchi K., Miyata N., Watanabe T. (1997). Immunologic and virologic characterization of the primary infiltrating cells in the aqueous humor of human T-cell leukemia virus type-1 uveitis. Accumulation of the human T-cell leukemia virus type-1-infected cells and constitutive expression of viral and interleukin-6 messenger ribonucleic acids. Investig. Ophthalmol. Vis. Sci..

[108] Mochizuki M., Watanabe T., Yamaguchi K., Takatsuki K., Yoshimura K., Shirao M., Nakashima S., Mori S., Araki S., Miyata N. (1992). HTLV-I uveitis: A distinct clinical entity caused by HTLV-I. Jpn. J. Cancer Res..

[109] Masuoka K., Sagawa K., Mochizuki M., Oizumi K., Itoh K. (1995). Polyclonal use of T-cell receptor alpha for human T-cell lymphotropic virus type 1-infected T cells. Investig. Ophthalmol. Vis. Sci..

[110] Sagawa K., Mochizuki M., Masuoka K., Katagiri K., Katayama T., Maeda T., Tanimoto A., Sugita S., Watanabe T., Itoh K. (1995). Immunopathological mechanisms of human T cell lymphotropic virus type 1 (HTLV-I) uveitis. Detection of HTLV-I-infected T cells in the eye and their constitutive cytokine production. J. Clin. Investig..

[111] Siverio-Llosa C., Silva-Ocas I., Gálvez-Olórtegui T., Arana-Kaik G. (2022). Clinical course of HTLV-1 infection associated intermediate uveitis. Arch. Soc. Esp. Oftalmol. (Engl. Ed.).

[112] La Grenade L., Manns A., Fletcher V., Carberry C., Hanchard B., Maloney E.M., Cranston B., Williams N.P., Wilks R., Kang E.C. (1998). Clinical, pathologic, and immunologic features of human T-lymphotrophic virus type I-associated infective dermatitis in children. Arch. Dermatol..

[113] De Oliveira M.D.F.S.P., Fatal P.L., Primo J.R.L., da Silva J.L.S., Batista E.D.S., Farré L., Bittencourt A.L. (2012). Infective dermatitis associated with human T-cell lymphotropic virus type 1: Evaluation of 42 cases observed in Bahia, Brazil. Clin. Infect. Dis..

[114] Tsukasaki K., Yamada Y., Ikeda S., Tomonaga M. (1994). Infective dermatitis among patients with ATL in Japan. Int. J. Cancer.

[115] Bittencourt A.L., Oliveira M.D.F., Brites C., Van Weyenbergh J., Vieira M.D.G.D.S., Araújo I. (2005). Histopathological and immunohistochemical studies of infective dermatitis associated with HTLV-I. Eur. J. Dermatol..

[116] Mahe A., Chollet-Martin S., Gessain A. (1999). HTLV-I-associated infective dermatitis. Lancet.

[117] Schierhout G., McGregor S., Gessain A., Einsiedel L., Martinello M., Kaldor J. (2020). Association between HTLV-1 infection and adverse health outcomes: A systematic review and meta-analysis of epidemiological studies. Lancet Infect. Dis..

[118] Valcarcel B., Enriquez-Vera D., De-La-Cruz-Ku G., Chambergo-Michilot D., Calderón-Huaycochea H., Malpica L. (2023). Epidemiological Features and Outcomes of HTLV-1 Carriers Diagnosed with Cancer: A Retrospective Cohort Study in an Endemic Country. JCO Glob. Oncol..

[119] Yamanashi H., Koyamatsu J., Nagayoshi M., Shimizu Y., Kawashiri S.-Y., Kondo H., Fukui S., Tamai M., Sato S., Yanagihara K. (2018). Human T-Cell Leukemia Virus-1 Infection Is Associated with Atherosclerosis as Measured by Carotid Intima-Media Thickness in Japanese Community-Dwelling Older People. Clin. Infect. Dis..

[120] Barr R.S., Drysdale S.B., Boullier M., Lyall H., Cook L., Collins G.P., Kelly D.F., Phelan L., Taylor G.P. (2022). A Review of the Prevention of Mother-to-Child Transmission of Human T-Cell Lymphotrophic Virus Type 1 (HTLV-1) with a Proposed Management Algorithm. Front. Med..

[121] Rosadas C., Taylor G.P. (2022). Current Interventions to Prevent HTLV-1 Mother-to-Child Transmission and Their Effectiveness: A Systematic Review and Meta-Analysis. Microorganisms.

[122] Yoshimitsu M., White Y., Arima N. (2014). Prevention of human T-cell lymphotropic virus type 1 infection and adult T-cell leukemia/lymphoma. Recent Results Cancer Res..

